# Early metabolic changes in the brain of Alzheimer’s disease rats are driven by GLAST+ cells

**DOI:** 10.1177/0271678X251318923

**Published:** 2025-02-07

**Authors:** William J Morrey, Kelly Ceyzériat, Quentin Amossé, Aurélien M Badina, Ben Dickie, Ingo Schiessl, Stergios Tsartsalis, Philippe Millet, Hervé Boutin, Benjamin B Tournier

**Affiliations:** 1 Division of Neuroscience, School of Biological Sciences, Faculty of Biology, Medicine and Health, University of Manchester, Manchester, UK; 2 Geoffrey Jefferson Brain Research Centre, Manchester Academic Health Science Centre, Northern Care Alliance & University of Manchester, Manchester, UK; 3 CIBM Center for BioMedical Imaging, Geneva, Switzerland; 4 Department of Psychiatry, University of Geneva, Geneva, Switzerland; 5 Department of Psychiatry, University Hospitals of Geneva, Geneva, Switzerland; 6 Department of Fundamental Neuroscience, 27213 University of Lausanne, Lausanne, Switzerland; 7 Imaging Brain & Neuropsychiatry iBraiN U1253, Université de Tours, Inserm, Tours, France

**Keywords:** Alzheimer’s disease, astrocyte-neurone lactate shuttle, brain metabolism, FDG-PET, MACS-RTT

## Abstract

Glucose metabolic dysfunction is a hallmark of Alzheimer’s disease (AD) pathology and is used to diagnose the disease or predict imminent cognitive decline. The main method to measure brain metabolism *in vivo* is positron emission tomography with 2-Deoxy-2-[^18^F]fluoroglucose ([^18^F]FDG-PET). The cellular origin of changes in the [^18^F]FDG-PET signal in AD is controversial. We addressed this by combining [^18^F]FDG-PET with subsequent cell-sorting and γ-counting of [^18^F]FDG-accumulation in sorted cell populations. 7-month-old male TgF344-AD rats and wild-type controls (n = 24/group) received sham or ceftriaxone (200 mg/kg) injection prior to [^18^F]FDG-PET imaging to increase glutamate uptake and glucose utilisation. The same animals were injected again one week later, and radiolabelled brains were dissected, with hippocampi taken for magnetically-activated cell sorting of radioligand-treated tissues (MACS-RTT). Radioactivity in sorted cell populations was measured to quantify cell-specific [^18^F]FDG uptake. Transcriptional analyses of metabolic enzymes/transporters were also performed. *Hypo*metabolism in the frontal association cortex of TgF344-AD rats was identified using [^18^F]FDG-PET, whereas *hyper*metabolism was identified in the hippocampus using MACS-RTT. Hypermetabolism was primarily driven by GLAST+ cells. This was supported by transcriptional analyses which showed alteration to metabolic apparatus, including upregulation of hexokinase 2 and altered expression of glucose/lactate transporters. See Figure 1 for summary.

## Introduction

Alzheimer’s disease (AD) is the most prevalent neurodegenerative disease, affecting 3–4% of people over 60 years old in Europe, Asia and America,^1^ with ageing populations driving increasing global incidence.

Brain glucose hypometabolism is a well-described characteristic of AD. The specific spatiotemporal progression of hypometabolism in the brain can be used to predict imminent cognitive decline from cognitively normal to mild cognitive impairment (MCI), and from MCI to AD.^2,3^ There may also be an early hypermetabolic phase driven by network disinhibition and/or glial reactivity, although the mechanisms and significance of this are still unclear.^4–13^ Understanding the mechanisms of early metabolic dysfunction therefore appears fundamental to understand the early drivers of AD pathology, and it remains to be determined whether they are a cause or consequence of neurodegeneration.

The gold standard method for imaging brain glucose metabolism *in vivo* is 2-Deoxy-2- [^18^F]fluoroglucose ([^18^F]FDG) which uses the ^18^F-radiolabelled glucose analogue, 2-deoxyglucose, to measure the rate of glycolysis. Like glucose, FDG can be transported across the blood-brain barrier (BBB) and cell membranes via GLUT transporters. FDG is also a viable substrate to the first glycolytic enzyme (hexokinase). However, unlike glucose, phosphorylated FDG cannot undergo further metabolism, and accumulates in cells at a rate proportional to uptake and phosphorylation.^14,15^ Whilst [^18^F]FDG-PET is highly sensitive, it lacks cellular specificity. The cellular source of the [^18^F]FDG-PET signal is contentious. Initial assumptions were that the signal was primarily dependent on neuronal activity. However, the development of the astrocyte-neurone lactate shuttle (ANLS) hypothesis has challenged this.^16^ Briefly, this hypothesis stipulates that glucose is preferentially taken into astrocytes via GLUT1, rather than directly into neurones via GLUT3 transporters. Glucose is then metabolised into lactate in astrocytes and subsequently shuttled via monocarboxylate transporters (MCTs) into neurons.^17^ This compartmentalisation means glycolysis is dominant in astrocytes, and mitochondrial oxidative phosphorylation prevails in neurones. The ANLS is driven by neuronal activity via mechanisms including glutamate uptake through GLT-1 transporters^17^ to support action potentials and neurotransmission. Given that the [^18^F]FDG-PET signal is proportional to the rate of FDG uptake and phosphorylation, this implies that the signal may be derived from astrocytes^18^ and, therefore, that the reduced metabolism seen in AD patients may also result from astrocytic/ANLS dysfunction, not only from neuronal dysfunction. It is precisely this relative contribution of each cell type to the [^18^F]FDG-PET signal that remains unclear.

Two recent independent studies combining [^18^F]FDG administration and magnetically activated cell sorting (a technique we will refer to here as ‘magnetically-activated cell sorting of radioligand-treated tissues’, or MACS-RTT) to assess cell-specific contributions to the [^18^F]FDG signal. These studies found that microglia showed the greatest accumulation of [^18^F]FDG in mouse models of AD,^5^ and that microglial activation can increase brain metabolism, as observed using [^18^F]FDG-PET in those models.^5,19^ These findings suggest we may need to revise our theories of brain metabolism, which often overlook microglia due to their relative lack of abundance in the brain.^20^ However, both studies used the same technique, so it is possible the high signal measured in microglia results from an artefact relating to MACS-RTT methodology. As of yet, investigations into cell-specific [^18^F]FDG accumulation have been performed in mouse models.^5^ Whilst mice are the most common models used for AD research, they are more distantly related to humans relative to rats and exhibit greater morphological and physiological divergence as a result. Hence, rat models of AD may more accurately reflect clinical pathology. The TgF344-AD rat model expresses familial AD mutations (APP_Swe_ and PS1_ΔE9_) and displays all the main pathophysiological features of AD such as age-dependent amyloid and tau pathology, neuroinflammation, alterations of brain connectivity, and cognitive impairments.^21–28^ Data on brain metabolism in TgF344-AD rats are however scarce. One study using chemical exchange saturation transfer MRI found significant reductions in brain glucose uptake by 9-months of age,^29^ implying that metabolic changes occur early in pathology in the strain, during early plaque formation, and an [^18^F]FDG-PET study found metabolic alterations at 14–16 months.^30^ This contrasts the evidence of hypermetabolism in other models and may reflect differences between models, methodology and/or disease stage. Here we present the first study investigating early brain metabolic changes in the TgF344-AD model using [^18^F]FDG-PET combined with cell sorting and transcriptional analyses.

The first aim was to identify early differences in metabolic rates in young TgF344-AD rats as compared to wild-type controls and to determine cell populations involved in those early changes. To address these questions, we measured brain metabolism in the brain using *in vivo* PET imaging and subsequent *ex vivo* γ-counting on sorted cell populations from the hippocampus in the same animals. Then, to explore mechanisms underlying the changes in metabolic rate, the expression of key glucose metabolic and transport mediators in cell populations was analysed by qPCR.

## Methods

### Animals

All experiments were approved by the Ethics Committee for Animal Experimentation of the Canton of Geneva, Switzerland, in accordance with Article 18 of the national law on animal protection (RS 455), Article 141 of the ordinance on animal protection (RS 455.1), and Article 30 of the ordinance on animal experimentation (RS 455.163). Experiments were reported in compliance with the ARRIVE guidelines (Animal Research: Reporting *in Vivo* Experiments) (https://www.nc3rs.org.uk/arrive-guidelines). A previous study showed 23% of variability in [^18^F]FDG uptake and a 14% increase in response to ceftriaxone.^31^ Thus, to observe less than 12% of difference with p ≤ 0.05 and β < 0.2 considering the [^18^F]FDG uptake, a sample size analysis performed using G*power (v3.1) indicated that the total sample size must be at least 46; we used 48 animals.

Male 7-month-old TgF344-AD rats (n = 24) and their age-matched wild-type counterparts (n = 24) were bred in-house and group-housed in 12-hour dark light cycles, with food and water *ad libitum*. Animals were fasted overnight prior to injection of [^18^F]FDG for both PET and MACS experiments to normalise blood glucose. All experiments were performed in the light phase.

Each genotype was randomly subdivided into Sham and Ceftriaxone-treated groups (n = 12 per group). Ceftriaxone-treated animals received a dose of 200 mg/kg ceftriaxone (Labatec Pharma SA, I.V.) immediately before each injection of [^18^F]FDG, to enhance astrocytic glutamate uptake and increase glucose utilisation, whilst Sham animals received an equal volume of injectable saline. Injections were performed intravenously to the lateral tail vein. Glycaemia of tail vein blood was measured immediately prior to each injection of [^18^F]FDG using a glucometer (CareSens® N Premier, 3401060164515).

PET and cell sorting experiments were each performed over 4 experimental days. For both PET and cell sorting, the permutation of groups was systematically randomised to nullify any effects of time of day on overall data (i.e. order of groups on Day 1: WT-Sham, WT-Cef, Tg-Sham, Tg-Cef; Day 2: Tg-Sham, Tg-Cef, WT-Sham, WT-Cef etc.). The main experimenter was blinded until groupwise analyses were performed. For both PET and cell sorting experiments, injection of [^18^F]FDG and ceftriaxone was performed under isoflurane anaesthesia. The duration of anaesthesia was kept short (<5 min) to minimise anaesthetic exposure and allow [^18^F]FDG to accumulate in the brain during wakefulness. During all periods of anaesthesia, body temperature was maintained using heated (37°C) beds.

### [^18^F]FDG PET

Animals (7 months old, WT bodyweight = 438.2 ± 19.9 g, Tg bodyweight = 488.8 ± 32.7 g) were anaesthetised with isoflurane (4% induction, maintained at 2%) in oxygen. [^18^F]FDG (injected dose: 25.36 ± 1.71 MBq) was injected via a tail vein catheter 40 minutes prior to imaging. The duration of anaesthesia for the injection of [^18^F]FDG and saline/ceftriaxone did not exceed 5 min. The rats were immediately awakened for [^18^F]FDG accumulation, during which, the animal was allowed to recover from anaesthesia and behave normally for 40 min until being re-anaesthetised for imaging. A CT image was acquired prior to PET acquisition. A static PET acquisition was performed for 20 minutes per animal, during which body temperature was maintained using a rectal probe and heated bed. Images were acquired on a FLEX TriumphTM preclinical PET-CT scanner (Gamma Medica-Ideas, Nortridge, CA).

[^18^F]FDG-PET images were spatially normalised to an [^18^F]FDG-PET template, which was registered to a T2-weighted MRI atlas using PMOD (v4.401, PMOD Technologies LLC). The registered images were then analysed using Statistical Parametric Mapping (SPM12) with the Small Animal Molecular Imaging Toolbox (SAMIT; v3.0) to generate parametric maps of standard uptake values (SUV) corrected for blood glucose, injected dose and weight_._ Relative SUV (SUVR) images were then generated using the cerebellum as a reference as it showed the lowest between-group variability. These were then analysed at the volume of interest (VOI) level in 30 VOIs in both hemispheres using PMOD to obtain bilateral regional SUVR averages.

### Magnetically-activated cell sorting of radioligand-treated tissues (MACS-RTT)

The same animals used for *in vivo* imaging were used for MACS-RTT. MACS-RTT was performed one week after PET imaging (WT bodyweight = 454.6 ± 30.0 g, Tg bodyweight = 503.2 ± 38.6 g). Anaesthesia was induced and maintained using isoflurane in oxygen at the same concentrations as during PET imaging. Anaesthetised rats were administered with [^18^F]FDG (injected dose: 56.68 ± 9.90 MBq) and either ceftriaxone or saline as described above. Immediately after injection, animals were returned to their home cage and the cage was moved to a separate shielded room to prevent excess exposure of experimenters to radiation. Animals were allowed to recover and were left awake for one hour before being re-anaesthetised and culled by guillotine. Brains were removed and the hippocampus was removed from one hemisphere. Each hippocampus was immediately weighed, then kept in ice-cold HBSS^−/−^ (Gibco, 14175095) temporarily (<20 minutes) until dissociation for cell sorting. The hippocampus was selected *a priori*, before having the results of the PET imaging, as it is well-described in the literature that the hippocampus is heavily and one of the first affected by the AD pathology in this model.^21,25^

Cells from hippocampal samples were dissociated using a neural tissue dissociation kit (Miltenyi Biotec, 130-094-802) according to manufacturer instructions. Samples were transferred to a proprietary dissociation solution and cut into small pieces before the suspensions were dissociated using a GentleMACS™ Octo Dissociator (Miltenyi Biotec, 130-096-427) according to manufacturer instructions. Cells were suspended in 400 µL of sorting buffer (0.5% BSA in PBS) and 100 µL myelin removal beads (Miltenyi Biotec, 130-096-733) per sample and separated into myelin-positive and myelin-negative cells using a MultiMACS™ Cell24 Separator Plus (Miltenyi Biotec, 130-098-637). Myelin-positive cells were discarded. Myelin-negative cells were centrifuged (5 min, 4°C, 300 g), the pellets were resuspended, and Fc receptors were blocked using an anti-rat CD32 (BD Bioscience, 550271). Then, cells were labelled for GLAST using a biotinylated anti-GLAST MicroBead Kit (Miltenyi Biotec, 130-095-826) according to manufacturer instructions and sorted in GLAST+ and GLAST- cells using the MultiMACS™ Cell24 Separator Plus. GLAST- cells were incubated with anti-rat CD11b/c MicroBeads (Miltenyi Biotec, 130-105-634) according to manufacturer instructions and sorted into CD11b+ and CD11b- populations. The resultant GLAST-/CD11b- population is herein referred to as unlabelled.

All populations were collected by centrifugation (5 min, 4°C, 300 g) and resuspended in 400 µL of Trizol (Thermofisher, 15596026), then radioactivity was immediately measured using an automatic γ-counter (Perkin Elmer, 2470-0050). The radioactivity measured by the gamma counter in the sorted cells was always greater than 10x the detection limit. The measured value was corrected for decay, injected dose, weight of the tissue sample, weight of the animal and glycaemia. To correct for day-to-day variability in the quality of tissue dissociation, these values were further normalised to the value from the WT Sham group for each cell type on each day. Samples were frozen at −80°C until later use. For full details of dissociation and cell sorting methods, see Supplementary Information

### RNA extraction and cDNA synthesis

RNA analysis was performed on the cell populations derived from the MACS-RTT experiment.

The cell populations in Trizol were defrosted and mixed with 100 µL chloroform then allowed to rest for 2–3 minutes prior to centrifugation (15 min, 4°C, 12 000 g). The aqueous phase was mixed with an equal volume of 70% ethanol. RNA extraction was then performed using a RNeasy™ Micro Kit (Qiagen, 74004) according to manufacturer instructions.

To synthesise first-strand cDNA, each sample of RNA (10 µL) was added to 1 µL of random primers (Thermofisher, 48190011) and 1 µL of 10 mM dNTP mix (Thermofisher, 10297018). The mixture was heated to 65°C for five minutes in a thermal cycler (ProFlex PCR System, Thermofisher, 4484073) before cooling on ice. Then, 4 µL of 5× First Strand Buffer (Thermofisher, Y02321), 2 µL of 100 mM DTT (Thermofisher, P2325) and 40 units of RNasin™ ribonuclease inhibitor (Thermofisher, N251A). The solution was incubated at 37°C before addition of 200 units of M-MLV Reverse Transcriptase (Thermofisher, 28025013). This solution was incubated at 25°C for 10 minutes, then 37°C for 50 minutes, then 70°C for 15 minutes in the thermal cycler. The samples were then immediately cooled to 4°C, diluted ten-fold in nuclease-free sterile water and stored at −20°C until use.

### qPCR

Primers (Supplementary Table 1) were designed using NCBI Blast tools (https://www.ncbi.nlm.nih.gov/). All primers were validated in titration experiments to determine specificity and efficiency prior to use in this study. This validation was performed on a series of four ten-fold dilutions of cDNA derived extracted from TgF344-WT brain tissue. Each dilution was measured in duplicate, with nuclease-free sterile water as a negative control for each primer.

qPCR experiments were performed using a QuantStudio™ 5 Real-Time qPCR Instrument (Thermofisher, A28140). All plates were filled using a Myra Liquid Handling System (Bio Molecular Systems, MYRA-LHS50). Each well contained 2 µL of sample cDNA and 8 µL of master mix, containing relevant primers, PowerUp™ SYBR™ Green (Thermofisher, A25779) and nuclease-free sterile water. The qPCR reaction included an enzyme activation step (95°C, 2 min), followed by 40 cycles of denaturation (95°C, 15 sec) and annealing (60°C, 1 min). C_q_ values were extracted using Quantstudio qPCR Design and Analysis Software (Thermofisher, v2.6.2) and converted to relative mRNA expression using the ΔΔC_t_ method with *Ppia* as the housekeeping gene.

### Data analysis and statistics

All data were analysed using Graphpad Prism v9.5.1 (GraphPad Software, Boston, Massachusetts USA, www.graphpad.com). All statistical tests were performed with a significance threshold of p = 0.05. In all tests, normality was assessed using the Kolmogorov-Smirnov test; normally distributed data were analysed with appropriate parametric tests, whereas non-normal data were analysed with non-parametric tests.

PET scans were analysed by three-way ANOVA, with Strain, ceftriaxone treatment and VOI as factors. A significant effect of VOI, but neither strain nor ceftriaxone effect nor interactions between these factors justified subsequent analysis, ceftriaxone-treated and sham controls within each strain were therefore pooled.

MACS-RTT and qPCR data were analysed by two-way ANOVA to assess strain and ceftriaxone effects. Whenever ceftriaxone was found to have no effect, sham and ceftriaxone-treated animals were pooled to analyse strain effects via *t*-tests or Mann-Whitney tests as appropriate. All data are expressed as mean ± standard deviation.

## Results

### [^18^F]FDG PET shows no overall change in [^18^F]FDG accumulation in Tg rats

Seven month-old rats were imaged with [^18^F]FDG-PET to quantify brain metabolism early in pathology. We administered ceftriaxone to enhance glucose utilisation. However, there was no effect of ceftriaxone in any brain region (Figure 2(a) and Supplementary Figure 1), so data from sham and ceftriaxone-treated groups were subsequently pooled to increase the power of our analyses.

**Figure 1.**
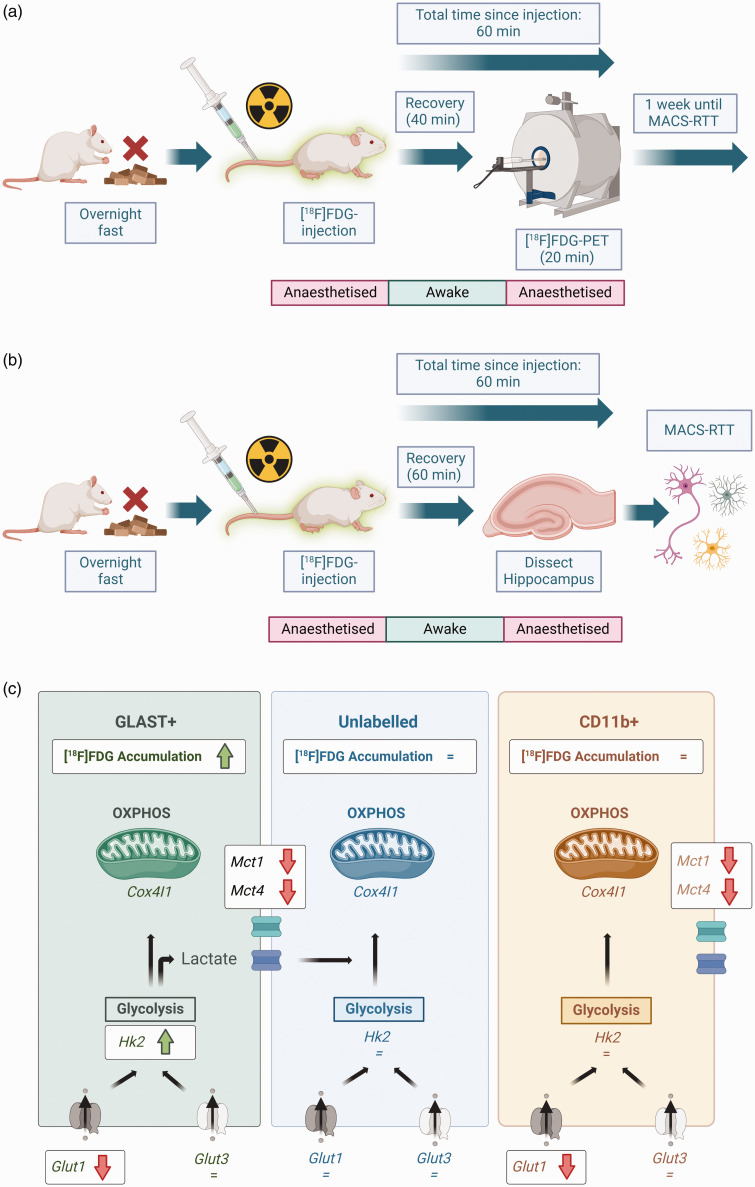
Overview of experimental design and effects of Alzheimer’s genotype on metabolism in different cell populations. (a) For [^18^F]FDG-PET imaging, animals were fasted overnight to normalise blood glucose levels prior to experimentation. Each animal then received intravenous [^18^F]FDG, and either ceftriaxone or saline. Animals were recovered for [^18^F]FDG accumulation to occur during wakefulness. Animals were re-anaesthetised and imaged using [^18^F]FDG-PET to measure brain metabolism *in vivo*. (b) One week after PET imaging, the same animals underwent MACS-RTT. The wakeful period was 60 min for MACS-RTT experiments (compared with 40 min for PET), to ensure the same total duration between tracer injection and the end of each experiment (PET scan duration was 20 min). After [^18^F]FDG accumulation, animals were re-anaesthetised and immediately culled. Hippocampal tissue was then sorted and analysed via MACS-RTT and (c) Hippocampal GLAST+ cells were hypermetabolic in TgF344-AD rats relative to WT. There were no genotype effects in the other two cell populations. GLAST+ cells were also the only cell population in which *Hk2* was upregulated, indicating higher glycolytic activity, in TgF344-AD rats. There were profound genotype effects on expression of *Glut1* (not *Glut3*) and monocarboxylate transporter RNA expression, indicating substantial differences in glucose and lactate uptake and shuttling in AD rats. Black arrows indicate progression of glucose, lactate or other downstream metabolic intermediates through metabolism. Created using BioRender (https://BioRender.com).

**Figure 2.**
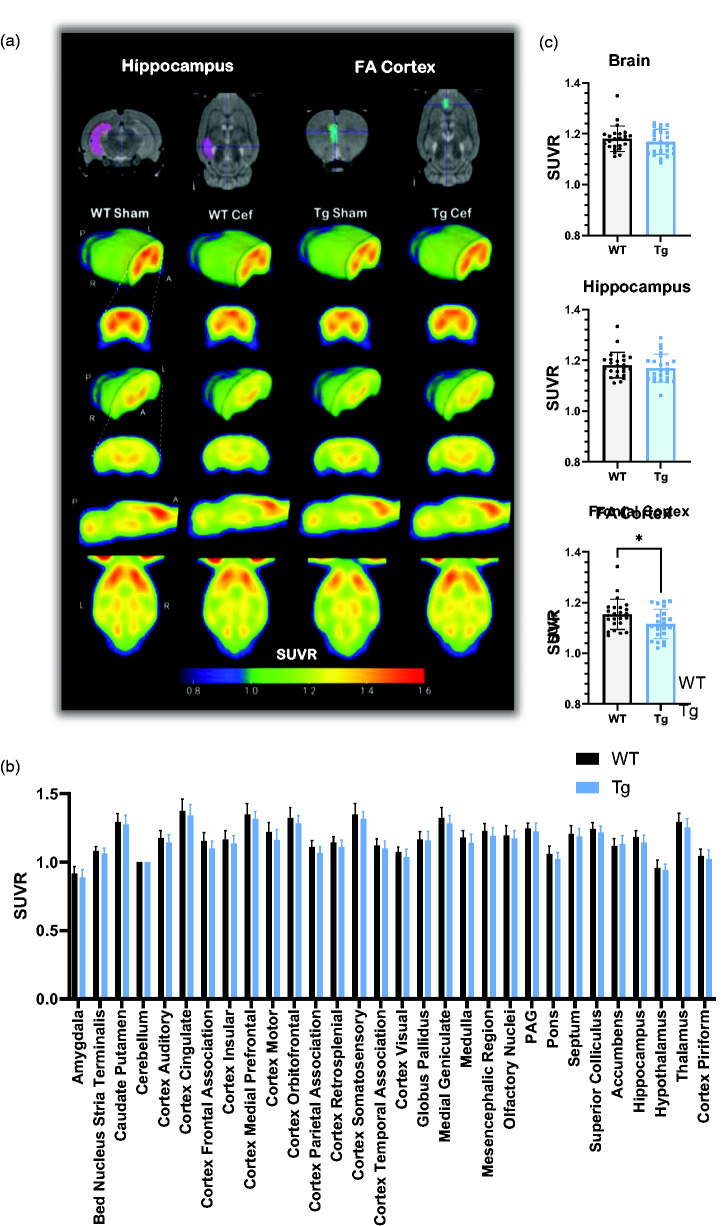
FDG-PET only identifies subtle metabolic changes in the frontal cortex between Tg and WT rats. (a) Averaged FDG-PET SUVR images for each group (n = 12), with MRI atlases demonstrating VOIs for key AD-related brain regions. (b) SUVR values for all VOIs after grouping sham and ceftriaxone-treated animals (n = 24). No differences were detected in any region when assessed via two-way ANOVA and (c) when assessing VOIs on an individual basis, no differences were detected in SUVR in the whole brain or hippocampus, but a small decrease was measured in the frontal cortex of Tg rats (unpaired two-tailed t-test, p = 0.034, n = 24). Data are shown as mean ± SD. Cef: ceftriaxone; FA: frontal association; PAG: periaqueductal grey.

The whole brain and individual VOIs SUVR values were not significantly different between genotypes when analysed with two-way ANOVA (Figure 2(b)). This was also true when individual VOIs were compared via *t*-test, with the exception of the frontal association cortex which was significantly but modestly lower in Tg (−3.3%) than WT rats (p = 0.034).

### MACS-RTT identifies increased [^18^F]FDG accumulation in Tg rats, mainly in GLAST+ cells

We then aimed to determine if there were differences in metabolic function between different cell populations in WT vs Tg rats .

Hippocampal cells were sorted to GLAST+, CD11b+ and unlabelled populations, with the intention to derive astrocyte-enriched, microglia-enriched, and neurone-enriched populations. RNA expression of common cell markers was assessed to characterise the sorted cell populations.

**Figure 3.**
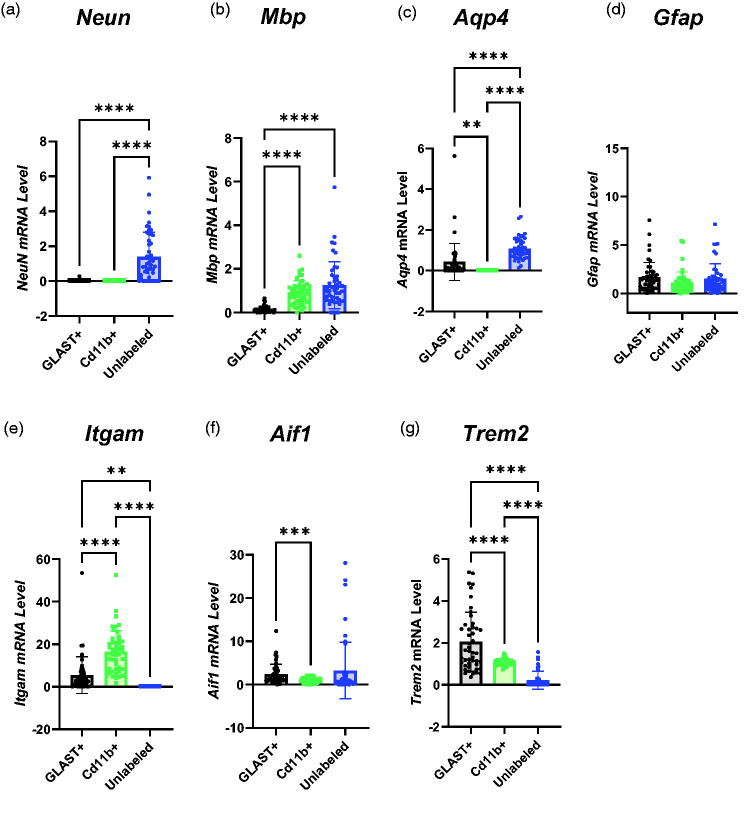
Characterisation of Sorted Cell Populations based on RNA Expression. (a) NeuN was expressed more in the unlabelled population than CD11b+ or GLAST+ cells (GLAST+ = 0.0088 ± 0.04, CD11b+ = 0.00097 ± 0.00042, Unlabelled = 1.39 ± 1.41). (b) Myelin basic protein (*Mbp*) RNA expression was higher in CD11b+ and Unlabelled cells than GLAST+ cells but not significantly different between CD11b+ and Unlabelled cells (GLAST+ = 0.15 ± 0.15, CD11b+ = 0.92 ± 0.54, Unlabelled = 1.25 ± 1.07). (c) No *Aqp4* expression was detected in CD11b+ cells, but it was higher in Unlabelled than GLAST+ cells (GLAST+ = 0.43 ± 0.90, CD11b+ = 00.00 ± 00.00, Unlabelled = 1.08 ± 0.53). (d) There were no differences in *Gfap* expression between cell populations (GLAST+ = 1.66 ± 1.53, CD11b+ = 1.08 ± 1.13, Unlabelled = 1.50 ± 1.55). (e) *Itgam* expression was higher in CD11b+ cells than GLAST+ cells. No *Itgam* expression was detected in the unlabelled population from any animal (GLAST+ = 5.42 ± 8.61, CD11b+ = 16.4 ± 9.9, Unlabelled = 0.00 ± 0.00). (f) *Aif1* RNA expression was higher in GLAST+ cells than CD11b+ cells but there were no other differences between groups (GLAST+ = 2.37 ± 2.32, CD11b+ = 1.02 ± 0.60, Unlabelled = 3.26 ± 6.51) and (g) *Trem2* expression was higher in GLAST+ cells than CD11b+ cells and higher in CD11b+ cells than Unlabelled cells (GLAST+ = 2.05 ± 1.41, CD11b+ = 0.72 ± 1.07, Unlabelled = 0.22 ± 0.43). All comparisons were made using one-way ANOVA (n = 24) and presented as mean ± SD. *Itgam*: integrin alpha M; *Neun*: neuronal nuclei; *Mbp*: myelin basic protein; *Aif1*: allograft inflammatory factor 1; *Trem2*: *triggering receptor expressed on myeloid cells 2*; *Aqp4*: *aquaporin 4.*

The canonical neurone marker *NeuN* (Figure 3(a)) was highly expressed in the unlabelled population, with a 158-fold increase relative to CD11b+ and 1433-fold increase relative to GLAST+ populations (p < 0.0001 in each case). Only 6 animals showed any expression of *Neun* in the CD11b+ or GLAST+ populations, and in each instance, this was very low (relative expression 0.028–0.28).

The expression of myelin basic protein (*Mbp*), a marker of oligodendrocytes, was similarly expressed in CD11b+ and unlabelled cells, although not in GLAST+ cells (Figure 3(b)).

No expression of *Aqp4*, an astrocytic marker, was detected in CD11b+ cells in any animal (Figure 3(c)), although it was expressed at a higher level in Unlabelled cells than GLAST+ cells (p < 0.0001). Another astrocyte marker, *Gfap* (Figure 3(d)), was expressed in all populations but showed no difference in expression between the three populations.

The canonical phagocytic cell marker *Itgam* (CD11b) was expressed three-fold more in CD11b+ cells relative to GLAST+ cells (Figure 3(e), p < 0.0001) and no *Itgam* expression was detected in the unlabelled population derived from any animal. However, another canonical microglial marker, *Aif1* (Iba1), showed 57% higher expression in the GLAST+ cells than the CD11b+ cells (p = 0.0009), and there were no other differences between populations (Figure 3(f)). *Trem2* (Figure 3(g)), a marker of microglia, was predominantly expressed in the GLAST+ population (two-fold higher than in CD11b+ cells and nine-fold higher than Unlabelled cells).

Given the lack of clear differentiation of cell types between the different populations, we have refrained from referring to cells as astrocytes, microglia, or neurones, and instead referred to them using the markers by which they were sorted.

**Figure 4.**
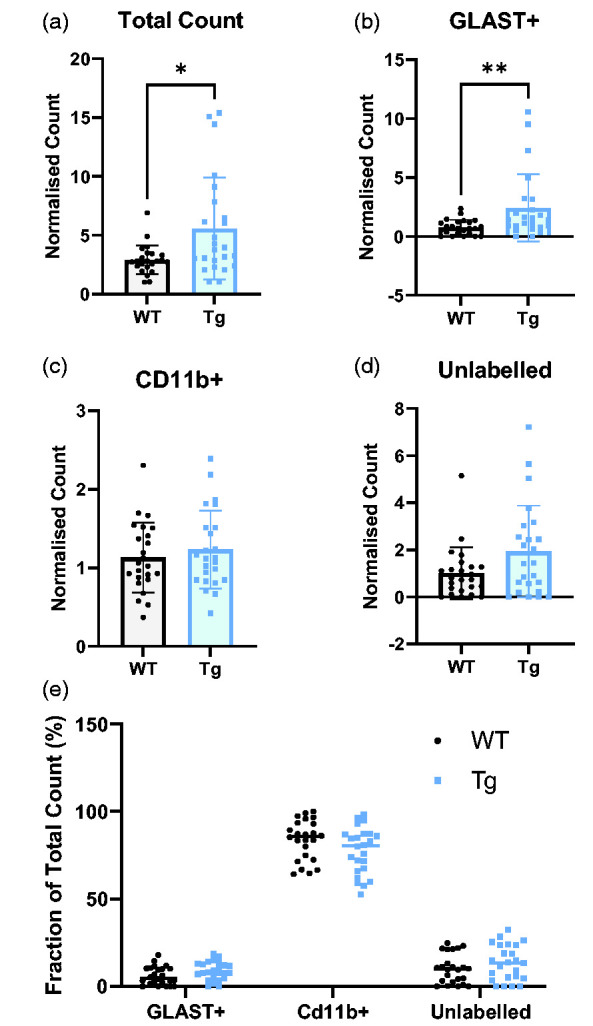
Tg brain cells have higher metabolic rate than WT - primarily driven by GLAST+ cells. CD11b+ cells have the greatest FDG-accumulation. (a) The total count, representing the sum of the normalised γ-counts of each cell population, is significantly higher in Tg rats (Tg = 5.58 ± 1.05, WT = 2.92 ± 0.12) (b–d) the normalised γ-count of GLAST+ cells was significantly higher in Tg rats (p = 0.0044). There were no significant differences in other cell populations, although there was a trend (p = 0.050) to increase in the unlabelled population. (e) The CD11b+ population has the greatest accumulation of FDG regardless of genotype (WT: 83.7% ± 11.3, Tg: 77.6% ± 13.3, p < 0.0001 vs GLAST+ and unlabelled cells). GLAST+ cells had the lowest uptake (WT: 6.1% ± 5.2, Tg: 8.9% ± 5.1) with unlabelled cells slightly higher (WT: 10.2% ± 8.3, Tg: 13.6%). Data were analysed using unpaired t-tests (n = 24) and presented as mean ± SD.

In contrast to the *in vivo* imaging data, we observed a two-fold increase in the [^18^F]FDG-accumulation in the pooled cellular populations of the hippocampus of Tg rats relative to WT (p = 0.016, Figure 4(a)). This increase was greatest in GLAST+ cells, which demonstrated a three-fold increase in [^18^F]FDG accumulation in Tg rats (p = 0.004, Figure 4(b), WT = 0.772 ± 0.20 vs Tg = 2.41 ± 0.37). In contrast, there was no difference in the metabolism of CD11b+ cells (Figure 4(c)) but there was a strong trend to increase in the unlabelled population (p = 0.05; Figure 4(d)).

Regarding the cellular origin of the [^18^F]FDG signal, we observed a significant higher accumulation of [^18^F]FDG in the CD11b+ population relative to the other cells (Figure 4(e)). These data are expressed as the percentage contribution of each cell type to the total count and, in both genotypes, CD11b+ cells contributed the vast majority to the total γ-count.

### qPCR reveals profound alterations to glycolytic and ANLS apparatus

**Figure 5.**
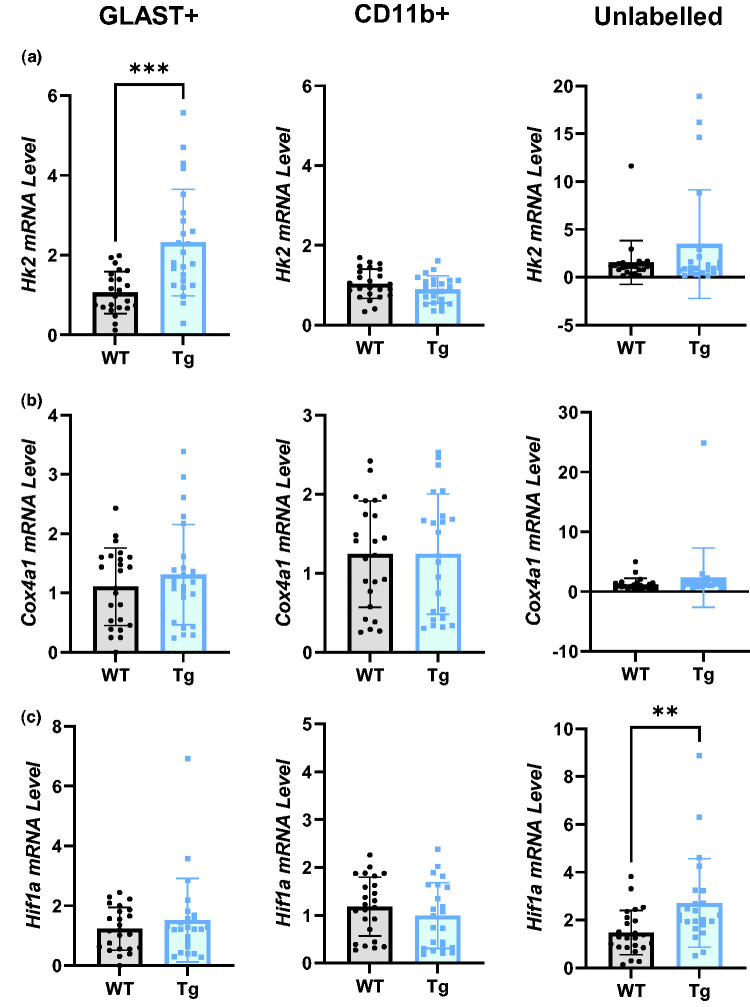
Glycolytic, but not oxidative, metabolic genes are upregulated in Tg rats. (a) Hexokinase 2 (Hk2) mRNA was significantly upregulated in GLAST+ cells of Tg rats, but not in CD11b+ or unlabelled cells. (b) *Cox4I1* (a key component in the electron transport chain) mRNA was not altered in any cell population in Tg rats vs WT and (c) *Hif1a* mRNA was upregulated in the unlabelled population, but not GLAST+ or CD11b+ populations, in Tg rats. All comparisons were made with unpaired t-tests (n = 24), data are presented as mean ± SD. Hk2: hexokinase 2; Cox4I1: Cytochrome oxidase C subunit 4 isoform 1; Hif1a: hypoxia inducible factor 1α.

The primary determinant of the rate of FDG-phosphorylation is hexokinase – the first enzyme and rate-limiting step in glycolysis. Here, we show that GLAST+ cells in Tg rats express 2.2-fold more *Hk2* than their WT counterparts (Figure 5(a); p = 0.0001, WT = 1.06 ± 0.53, Tg = 2.32 ± 1.34), whereas there was no difference between genotypes in CD11b+ (WT = 1.04 ± 0.37. Tg = 0.89 ± 0.34) and unlabelled cells (WT = 1.54 ± 2.28, Tg = 3.47 ± 5.67).

We measured no differences in the *Cox4a1*, a component of mitochondrial oxidative metabolism, between genotypes in any cell (GLAST+ WT =1.11 ± 0.65, Tg = 1.31 ± 0.85; CD11b+ WT = 1.24 ± 0.67, Tg =1.24 ± 0.76) (Figure 5(b)). Our data also suggest a shift towards a more glycolytic phenotype in the unlabelled population, indicated by a two-fold upregulation of mRNA for the metabolic regulator HIF-1α (Figure 5(c), p = 0.0026, WT = 1.48 ± 0.93, Tg = 2.7 ± 1.85). No differences in *Hif1a* expression were found in the GLAST+ (WT = 1.23 ± 0.29, Tg = 1.52 ± 0.61) or CD11b+ (WT = 1.18 ± 0.04, Tg = 0.99 ± 0.27) populations.

**Figure 6.**
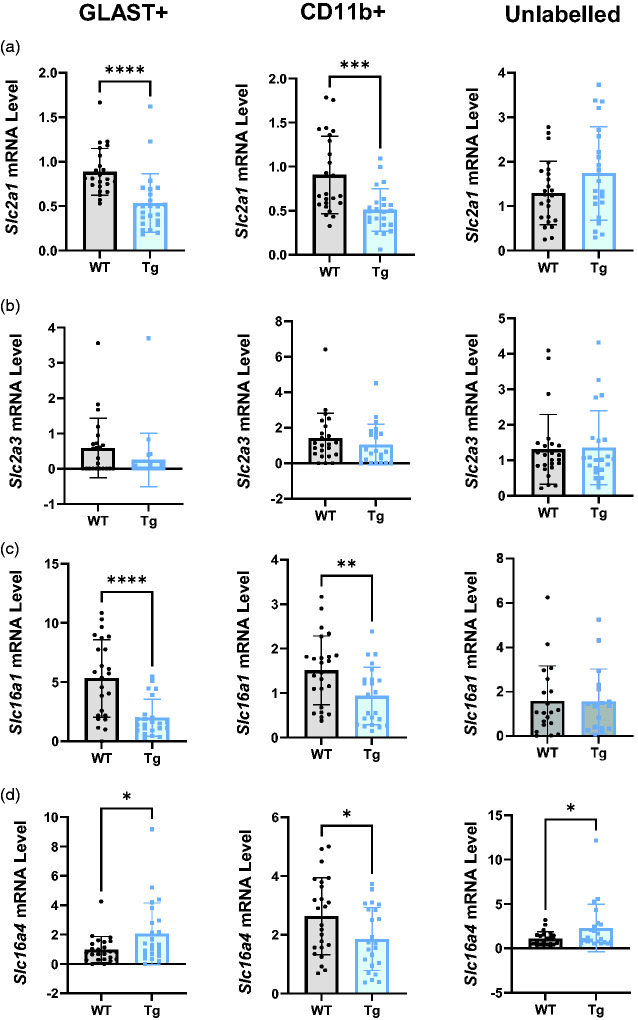
Transport apparatus for glucose uptake and lactate shuttling are profoundly altered in Tg rats. (a) *Slc2a1* mRNA was significantly lower in the GLAST+ and CD11b+ populations in Tg relative to WT rats. (b) *Slc2a3* mRNA expression was not altered in any cell population in Tg rats relative to WT. (c) *Slc16a1* mRNA expression was significantly reduced in GLAST+ and CD11b+ cells of Tg rats but unaltered in unlabelled cells and (d) *Slc16a4* mRNA was higher in GLAST+ and unlabelled cells in Tg rats. In CD11b+ cells, there was reduced expression of *Slc16a4* mRNA in Tg rats. All comparisons were made using unpaired t-tests (n = 24) and data are presented as mean ± SD. Slc2a1: glucose transporter 1; Slc2a3: glucose transporter 3; Slc16a1: monocarboxylate transporter 1; Slc16a4: monocarboxylate transporter 4.

*Glut1* was significantly down-regulated in Tg rats in both GLAST+ (Figure 6(a), p < 0.0001, WT = 0.89 ± 0.26, Tg = 0.53 ± 0.33) and CD11b+ cells (p = 0.0002, WT = 0.91 ± 0.44, Tg = 0.51 ± 0.24), although *Glut1* expression was unaltered in the unlabelled population (WT = 1.30 ± 0.71, Tg = 1.74 ± 1.05). In contrast, *Glut3* was not affected by the AD genotype in any population (Figure 6(b); GLAST+ WT = 0.59 ± 0.85, Tg = 0.25 ± 0.76; CD11b+ WT = 1.41 ± 1.40, Tg = 1.05 ± 1.14; Unlabelled WT = 1.31 ± 0.98, Tg = 1.36 ± 1.04).

Lactate shuttling apparatus was also profoundly affected (Figure 6(c) and (d)), with significant reductions in the expression of *Mct1* in GLAST+ (p < 0.0001, WT = 5.32 ± 3.27, Tg = 1.99 ± 1.56) and CD11b+ cells (p = 0.0084, WT = 1.51 ± 0.78, Tg WT = 0.94 ± 0.65), although there was no difference in the unlabelled population (WT = 1.59 ± 1.58, Tg = 1.57 ± 1.47). Similarly, there was a decrease in *Mct4* expression in the CD11b+ population (Figure 6(d), p = 0.032, WT = 2.63 ± 1.31, Tg = 1.86 ± 1.07), although it was upregulated in the GLAST+ (p = 0.048, WT = 0.98 ± 0.90, Tg = 2.05 ± 2.10) and unlabelled populations (p = 0.037, WT = 1.08 ± 0.79, Tg = 2.29 ± 2.67).

### Inflammatory markers are predominantly upregulated in GLAST+ cells

To investigate the role of glial activation and inflammation in the observed metabolic alterations early in pathology of TgF344-AD rats, we investigated key markers of glial reactivity and inflammatory cytokines.

**Figure 7.**
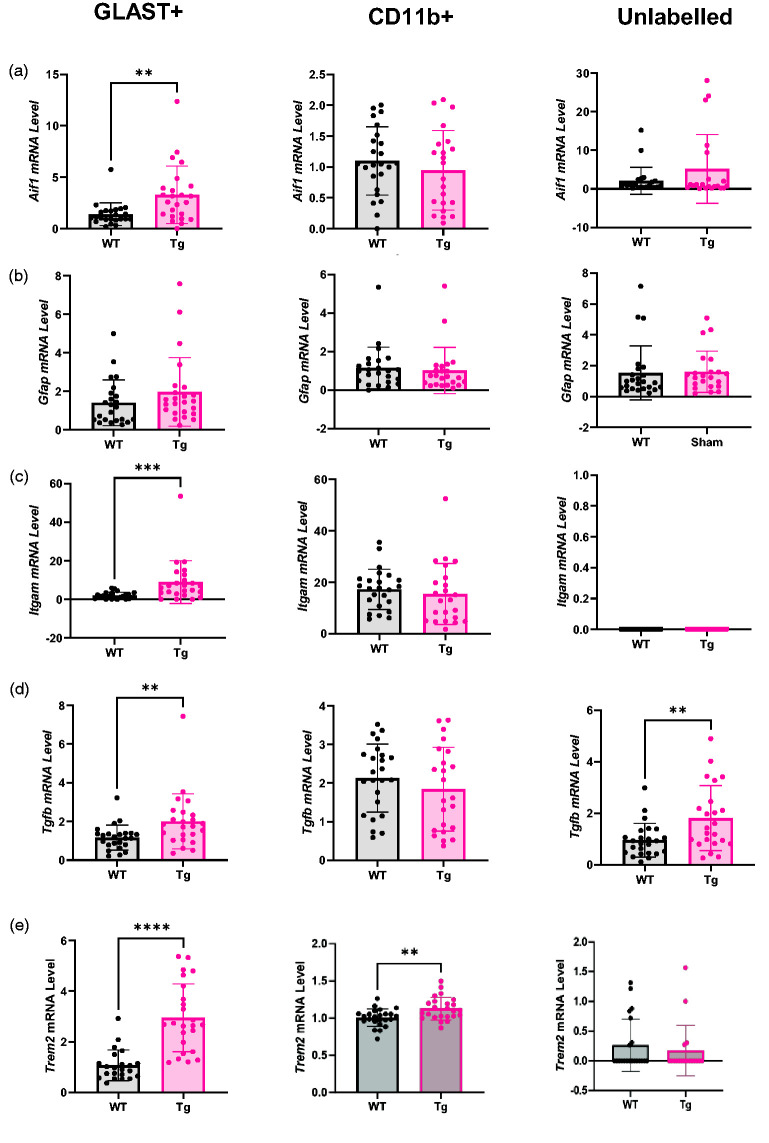
GLAST+ and unlabelled cells display inflammatory transcriptional changes in Tg rats. (a) *Aif1* mRNA was higher in the GLAST+ population, but not other cell populations, in Tg rats. (b) There was no difference in *Gfap* mRNA expression in any cell population. (c) *Itgam* mRNA was upregulated in the GLAST+ population but not in other populations in Tg rats. (d) *Tgfb* mRNA was higher in the GLAST+ and unlabelled cell populations of Tg rats and (e) *Trem2* was higher in Tg rats in GLAST+ cells and CD11b+ cells, but there was no difference between genotypes in the unlabelled population. All comparisons were made using two-tailed t-tests (n = 24) and expressed as mean ± SD. Aif1: allograft inflammatory factor 1; Gfap: glial fibrillary acidic protein; Itgam: integrin alpha M; Tgfb: transforming growth factor β; Trem2: triggering receptor expressed on myeloid cells 2.

A*if1* expression (Figure 7(a)) was two-fold higher in the GLAST+ cells of Tg rats (p = 0.002, WT = 1.40 ± 1.10, Tg = 3.30 ± 2.79) but there were no other differences (CD11b+ WT = 1.10 ± 0.55, Tg = 0.95 ± 0.64; Unlabelled WT = 2.10 ± 3.50, Tg = 5.17 ± 8.87).

No difference in *Gfap* expression was detected between WT and Tg in any cell population (Figure 7(b); GLAST+ WT = 1.41 ± 1.18, Tg = 1.96 ± 1.78; CD11b+ WT = 1.14 ± 1.09, Tg = 1.02 ± 1.20; Unlabelled WT = 1.53 ± 1.61, Tg = 1.61 ± 1.34).

*Itgam* expression was 4.6-fold higher in Tg relative to WT rats in the GLAST+ population (p < 0.001, WT = 1.96 ± 1.74, Tg = 8.96 ± 11.05) but there was no genotype effect in CD11b+ cells (WT = 17.28 ± 7.83, Tg = 15.48 ± 11.86) and no expression was detected in unlabelled cells (Figure 7(c)).

*Tgfb* expression was 71% higher in the GLAST+ population of Tg rats relative to WT rats (p = 0.005, WT = 1.17 ± 0.64, Tg = 2.00 ± 1.42) but not in the CD11b+ (WT = 2.13 ± 0.88, Tg = 1.84 ± 1.08) or Unlabelled (WT = 0.96 ± 0.65, Tg = 1.81 ± 1.26) populations (Figure 7(d)).

*Trem2* was 2.8-fold higher in Tg rats in GLAST+ cells (WT = 1.07 ± 0.60, Tg = 2.95 ± 1.34, p < 0.0001) and CD11b+ cells (WT = 1.01 ± 0.12, Tg = 1.13 ± 0.15, p = 0.003) (Figure 7(e)).

## Discussion

This study was the first to combine [^18^F]FDG-PET imaging with *ex vivo* γ-counting of Magnetically sorted cell populations to probe cell specific glucose metabolism and transport apparatus in the TgF344 model. We chose young (7 months) rats, an age which is early in relation to the evolution of the AD pathology in this model, but at which there are already increased levels of Aβ-42 oligomers, Triton soluble Aβ-40 and total Tau (sarkosyl-soluble and crude pellet).^21,25^ Using [^18^F]FDG-PET, we found a modest but significant decrease in glucose uptake in the frontal association cortex of Tg rats vs WT, a region only associated with hypometabolism in the later stages of clinical AD,^32^ but no differences in other regions. In contrast, our *ex vivo* analyses provide evidence of hippocampal hypermetabolism in an early stage of pathology driven by GLAST+ cells measured using MACS-RTT.

There is increasing awareness of a *hyper*metabolic phase in the earliest phases of amyloid-β fibrilisation preceding hypometabolism. This has been shown in cognitively normal humans,^4,7,10,11^ MCI patients,^8,12^ APOE ε4 carriers^11^ and familial AD mutation carriers,^6^ as well as mouse models of AD,^5,19^ and may be driven by a reactivity-induced increase in glial metabolism^4,5^ or network disinhibition.^13^ These hypermetabolic regions also appear to be susceptible to accelerated pathology at later stages in longitudinal imaging.^33^ Our data provide convincing evidence to support this hypermetabolic phase in early AD and are the first to do so in a rat model. Our data also show that hypermetabolism is driven predominantly by GLAST+ cells. The disparity between MACS-RTT and [^18^F]FDG-PET in the hippocampus implies higher sensitivity of the MACS-RTT technique. Due to logistical constraints, the hippocampus was selected *a priori*, before PET imaging data were analysed, as it is a region affected severely and early in AD pathology in this model.^21,25^ The discrepancy between MACS-RTT and [^18^F]FDG PET in the hippocampus is unlikely to be caused by the lack of cell type-discrimination in [^18^F]FDG-PET, as the total count of all populations in our MACS-RTT data remained significantly higher in the Tg group. The absence of genotype-related differences in [^18^F]FDG-PET imaging but presence of genotype differences in GLAST+ cells implies that the effect is too weak to be observed at regional tissue level. This increased sensitivity when measuring sorted cells confirms our previous observations that a difference between AD and control subjects only appeared after cell sorting.^24^ Two factors likely contribute to this: 1) amalgamation of multiple cell types in data derived from PET imaging ‘dilutes’ the genotype effects measured in GLAST+ cells that were not seen in other cell types, and 2) partial volume effects, which reduce PET sensitivity, particularly in areas in close proximity to CSF (e.g. the hippocampus), but do not affect MACS-RTT measurements. It is also possible that genotype differences measured using [^18^F]FDG-PET were diminished by 20 min of additional anaesthesia during the image acquisition and this may have reduced the effect size of differences between Tg and WT rats. We decided to control the overall duration of time after injection of [^18^F]FDG between MACS and PET (60 min). However, due to the 20 min acquisition period for PET, this meant a shorter waking period between injection/imaging and a longer period of anaesthesia during the PET experiment relative to the MACS-RTT experiment.

Our MASC-RTT data suggest that the early hypermetabolic phase is driven by GLAST+ cells, whereas earlier studies have highlighted microglia as the drivers of hypermetabolism in mice and patients.^4,5^ This discrepancy may reflect that we actually have a significant proportion of microglia in the GLAST+ population. Alternatively, if the GLAST+ population is truly enriched with astrocytes, the different findings between experiments may be explained by species or model specific differences or a combination of species and disease stage differences.

Whilst this enhanced metabolic rate is important, the extent and complexity of metabolic disturbances were also revealed here through transcriptional analyses, which show profound alterations to key enzymes for glycolytic metabolism and transporters for glucose and lactate. The upregulation of the key limiting glycolytic enzyme *Hk2* in GLAST+ cells is likely responsible for the observed hypermetabolism. This may reflect increased demands on glial cells attempting to maintain brain homeostasis during the early challenges of AD pathology. It has been shown that pathological astrocyte activation in the EAE model of multiple sclerosis reduces HK2 activity,^34^ so it is possible that the increase measured in our study represents anti-inflammatory activation of astrocytes, as supported by the increase in *Tgfb* and *Trem2* measured in GLAST+ cells. This is further supported by the neuroprotection and cognitive improvements following enhancement of astrocytic glycolysis^35^ and the increased amyloid accumulation caused by inhibiting astrocytic glycolysis^36^ in preclinical models of AD. Conversely, upregulated *Hk2* and glycolysis have been shown to be necessary for the expression of the pro-inflammatory response in both microglia and astrocytes,^37,38^ highlighting the heterogeneity of glial responses and the need for further research into the involvement of the metabolic processes in glial reactivity. We measured no alterations to *Cox4i1* expression, a marker of oxidative metabolism.

The mechanisms of transporting glucose and other energy metabolites are complex, involving uptake across the blood-brain barrier into different cells and shuttling of lactate from astrocytes to neurones by an array of glucose and monocarboxylate transporters.^17^ Here, we demonstrate extensive disruption to this network at the transcription level. The large decrease in *Slc2a1* expression in GLAST+ and CD11b+ cells is characteristic of reduced expression in AD^39,40^ and implies reduced capacity for glucose uptake into these cells. In terms of lactate transporters, there is an increase in *Mct4* but a larger decrease in *Mct1* expression in GLAST+ cells of Tg rats, which suggests reduced capacity for lactate transport to support neuronal function.^41,42^ This is exacerbated by the reduction of *Mct4* in the neurone-enriched unlabelled population, which would reduce lactate uptake from the extracellular space, and may underly the increased lactate concentration measured via magnetic resonance spectroscopy in the TgF344-AD model.^43^ MCTs play a role in modulating microglial reactivity so these changes may also contribute to the brain inflammatory response of young Tg rats.^44^

Our most controversial finding is that the population with greatest [^18^F]FDG accumulation was the CD11b+ population. This seemingly contradicts both of the two opposing arguments in the debate over whether astrocytes or neurones are primarily responsible for the majority of glucose utilisation, although this is difficult to conclude because of the limitations associated with our cell sorting. Whilst controversial, this finding is supported by the finding that microglia express more hexokinase than other major neural cells, regardless of activation status^45^ and strikingly similar results have been published in two other recent papers using different preclinical AD models.^5,19^ The latter study went on to show the prominent role of microglial activation state on brain metabolism in [^18^F]FDG-PET data. This indicates we may need to re-evaluate our current thinking on the relative contribution of different brain cells to metabolic imaging data, and the implications for understanding these data in the context of disease mechanisms. Nonetheless, it is important to note that these studies, like ours, combined MACS with γ-counting and, given the wealth of data from different modalities which support astrocytes (or possibly neurones) as the major contributors to glucose metabolism in human, rats and cell cultures^16–18,31,46–50^ this may imply that these findings may be influenced by methodological bias.

Some limitations of the MACS technique are discussed here in brief. Firstly, MACS gives no information about the number of cells in each population. This may explain the low γ-count in the GLAST+ population, since there are no universal astrocyte cell surface markers^51^ and therefore sorting cells in this manner will only yield a subpopulation of cells. As such, there may be a larger number of microglia relative to astrocytes, which would obscure the true per-cell glucose/FDG uptake and would not accurately reflect physiology since astrocytes are more numerous *in vivo.*^52^ However, a previous MACS-RTT experiment also performed cell counting to calculate the accumulation in individual cells, and microglia remained by far the highest contributor to FDG accumulation even when expressed per cell.^5^ Indeed, our whole population measure may more accurately reflect the contribution to imaging data than individual cell data, since microglia only represent a minority (∼7%) of brain cells^20^ and imaging data reflect tissular, not cellular values. Alternatively, MACS utilises one marker per population and is therefore an enrichment technique, rather than a true sorting method, and this has led to some unexpected results which indicate a mixture of cell types in each population. This may have been caused by a difference in the efficiency of antibodies to capture an entire cell population, and a resultant difference in the final cell count between GLAST+, CD11b+ and unlabelled cells. Thus, genotype differences for a given cell population are more relevant than comparisons between cell types without the addition of cell counting to provide per-cell γ-count values. The time between [^18^F]FDG injection and γ-counting (7–8 hours) may explain the differences in γ-counts if the rate of dephosphorylation of FDG-6-phosphate differs significantly between populations, as raised in a commentary on the Xiang *et al.* study^5,53^. The rate of dephosphorylation is described by the constant *k4* in compartmental kinetic models. Whilst *k4* has negligible effect in PET imaging for durations under 120 min,^15^ the extended time between injection and measurement presumably increases this contribution. As the brain’s cellular source of glycogen, astrocytes express the majority of enzymes for glycogenolysis, including glucose-6-phosphatase,^54^ which dephosphorylates glucose/FDG-6-P and would facilitate FDG efflux from cells. It is also possible that, in the absence of blood supply, astrocytes in suspension upregulate glycogenolytic apparatus and further enhance this dephosphorylation. However, the rate of enzymatic dephosphorylation is likely very low in cell preparations kept on ice, so it seems unlikely that this contribution would be large enough to account entirely for the difference between [^18^F]FDG accumulation in GLAST+ and CD11b+ populations measured here and in the other studies cited. Work should be done to characterise cell-specific FDG kinetics over long periods to clarify this.

We should also highlight that no effects of ceftriaxone were observed on metabolism *in vivo* or *ex vivo*, nor in the expression of *Slc1a2*, which is in contradiction with existing literature.^31^ This is likely due to the short time period between ceftriaxone injection and imaging/MACS-RTT, which may have been insufficient for upregulation of *Slc1a2* to occur. This short time interval was selected to minimise the duration of anaesthesia, which would have altered [^18^F]FDG uptake, but will be optimised in future work to allow us to modulate metabolic function with ceftriaxone. Nonetheless, the lack of ceftriaxone effect does not detract from the important genotype effects observed in this study.

In conclusion, our study clearly shows that GLAST+ cells are hypermetabolic early in pathology in the TgF344-AD model. GLAST+ cells also showed the greatest disturbances to glycolytic and lactate shuttling apparatus. In addition, these same cells are the ones showing upregulation of inflammatory markers. This points to the presence of enhanced mechanisms in the early stages of pathology primarily localised within GLAST+ cells that require further study. Of particular interest would be longitudinal investigations to assess whether upregulation of anti-inflammatory genes (e.g. *Trem2* and *Tgfb1*) becomes superseded by pro-inflammatory markers, and how this relates to the timecourse of metabolic pathology in the TgF344-AD model.

## Supplemental Material

sj-pdf-1-jcb-10.1177_0271678X251318923 - Supplemental material for Early metabolic changes in the brain of Alzheimer’s disease rats are driven by GLAST+ cells Supplemental material, sj-pdf-1-jcb-10.1177_0271678X251318923 for Early metabolic changes in the brain of Alzheimer’s disease rats are driven by GLAST+ cells by William J Morrey, Kelly Ceyzériat, Quentin Amossé, Aurélien M Badina, Ben Dickie, Ingo Schiessl, Stergios Tsartsalis, Philippe Millet, Hervé Boutin and Benjamin B Tournier in Journal of Cerebral Blood Flow & Metabolism
