# Supplementary material for: Early metabolic changes in the brain of Alzheimer’s disease rats are driven by GLAST+ cells
Source: J Cereb Blood Flow Metab. 2025 Feb 7;45(7):1326–42. doi: 10.1177/0271678X251318923 (PMC11806453; doi:10.1177/0271678X251318923)
Supplement: sj-pdf-1-jcb-10.1177_0271678X251318923 - Supplemental material for Early metabolic changes in the brain of Alzheimer’s disease rats are driven by GLAST+ cells [file sj-pdf-1-jcb-10.1177_0271678X251318923.pdf]

Detailed Methods

MACS-RTT

After using the GentleMACS™ Octo Dissociator, dissociation was completed by briefly centrifuging the samples at 4°C and gently pipetting the resultant pellet 25 times (5 × 1000 µL, 5 × 200 µL, 15 × 10 µL). Samples were filtered using 70 µm pre-separation filters (Miltenyi Biotec, 130-095-823) and gently washed with 10 mL HBSS$^{+/+}$ (Gibco, 11560616). After filtering, samples were centrifuged (5 min, 4°C, 600 g). After removal of supernatant, cells were suspended in 400 µL of sorting buffer (0.5 % BSA in PBS) and 100 µL myelin removal beads (Miltenyi Biotec, 130-096-733) per sample and left to incubate on ice for 15 minutes. Cells were washed in 5 mL of sorting buffer before centrifugation (10 min, 4°C, 300 g) and resuspension in 1 mL of sorting buffer. The resulting suspensions were then separated into myelin-positive and myelin-negative cells using a MultiMACS™ Cell24 Separator Plus (Miltenyi Biotec, 130-098-637), and myelin-positive cells were discarded.

Myelin-negative cells were centrifuged (5 min, 4°C, 300 g), the pellets were resuspended and Fc receptors were blocked using an anti-rat CD32 (BD Bioscience, 550271) for 10 min to avoid unspecific antibody binding. Then, cells were labelled for GLAST using a biotinylated anti-GLAST MicroBead Kit (Miltenyi Biotec, 130-095-826) according to manufacturer instructions. After labelling, GLAST-positive cells were separated using the GentleMACS™ Octo Dissociator. These cells were collected by centrifugation (5 min, 4°C, 300 g) and resuspended in 400 µL of Trizol (Thermofisher, 15596026), then radioactivity was immediately measured using an automatic γ-counter (Perkin Elmer, 2470-0050). Samples were frozen at -80°C until later use.

The remaining GLAST-negative cells were incubated with anti-rat CD11b/c MicroBeads (Miltenyi Biotec, 130-105-634) according to manufacturer instructions. The cells were

separated using the GentleMACS™ Octo Dissociator to obtain CD11b-positive cells (microglia) and CD11b-negative/GLAST-negative cells (unlabelled cells). Each cell population was centrifuged (5 min, 4°C, 300 g) and resuspended in 400 µL of Trizol before γ-counting and storage at -80°C.

All solutions and reagents were made fresh per day of cell-sorting and kept cold throughout the process.

qPCR

Full details of primers used in this study are provided in Table 1.

Table 1: Primer Details

Target	***Forward Primer***	***Reverse Primer***	***Efficiency (%)***	***R^2***
**Glucose Metabolism and Transport**				
**Hexokinase 2 (*Hk2*)**	5-CCAAGCGTCTCCATAAGGCA -3	5-GTTGGTCAGCCAGACGGTAA-3	126.8	0.99
**Cytochrome C oxygenase subunit 4I1 (*Cox4i1*)**	5-GAGTGGAAGACAGTGGTGGG -3	5-GGATGGGGCCATACACGTAG-3	98.9	0.99
**GLUT1 (*Slc2a1*)**	5-GCATCTTCGAGAAGGCAGGT-3	5-AACAGCGACACCACAGTGAA-3	190.5	0.99
**GLUT3 (*Slc2a3*)**	5-ATGGGGACAGCGAAGGTGAC-3	5-CAACCGCTCTTCCAACGTGT-3	156.4	0.97
**MCT4 (*Slc16a3*)**	5-CGATACTTCAACAAGCGCCG-3	5-CAGTGCACAAAGGAACACGG-3	132.7	0.98
**MCT1 (*Slc16a1*)**	5-GCTGCTTCTGTTGTTGCGAA-3	5-AAATCCAAAGACTCCCGCGT-3	105.3	0.99
**GLT-1 (*Slc1a2*)**	5-CCAACACCGAATGCACGAAG-3	5-TACGACAGAGTTGTGTGCGG-3	102.6	0.98

**HIF1α (*Hif1a*)**	5-AGCAATTCTCCAAGCCCTCC-3	5-CGGTGGCAGTGACAGTGAT-3	104.6	0.98
**Inflammation, Glial Reactivity and Cell Characterisation**				
**TGF-β (*Tgfb1*)**	5-CCTGGAAAGGGCTCAACAC-3	5-CAGTTCTTCTCTGTGGAGCTG-3	130.0	1.00
**CD11b (*Itgam*)**	5-CTTGGTGAAACCCGAGTGGT-3	5-TCGATCGTGTTGATGCTACCG-3	390.1	0.96
**GFAP (*Gfap*)**	5-TTGACCTGCGACCTTGAGTC-3	5-GAGTGCCTCCTGGTAACTCG-3	109.2	1.00
**MBP (*Mbp*)**	5-TCTCAGACCGCCTCAGAAGA-3	5-TGTGCTTGGAGTCTGTCACC-3	132.4	0.99
**Iba1 (*Aif1*)**	5-GCCAGAGCAAGGATTTGCAG-3	5-TGAAGGCCTCCAGTTTGGAC-3	132.5	1.00
**NeuN (*Fox3*)**	5-GAGTCTATGCGGCTGCTGAT-3	5-CAGAGGCTAGCCATGGTTCC-3	219.4	0.97
**TREM2 (*Trem2*)**	5-AACTTCAGATCCTACTGGACCC -3	5-GCAGAACAGAAGTCTTGGTGG -3	148	1.00
**AQP4 (*Aqp4*)**	5-TGGGAGTCACCACGGTTCAT-3	5-TCACAGCTGGCAAAAATGGTG-3	186	0.97
**Housekeeping Gene**				
**Cyclophilin A (*Ppia*)**	5-ATGGCAAATGCTGGACCAAA-3	5-GCCTTCTTTCACCTTCCCAAA-3	178	1.00

Abbreviations: GLUT1 = glucose transporter 1; GLUT3 = glucose transporter 3; MCT4 = monocarboxylate transporter 4; MCT1 = monocarboxylate transporter 1; GLT-1 = glutamate transporter 1; HIF1α = hypoxia-inducible factor 1α; TGF-β = transforming growth factor β; CD11b = cluster of differentiation molecule 11b; GFAP = glial fibrillary acidic protein; MBP = myelin basic protein; Iba1 = ionised calcium binding adaptor molecule 1; NeuN = neuronal nuclei; TREM2 = triggering receptor expressed on myeloid cells 2; Aqp4 = aquaporin 4.

Results

Ceftriaxone did not affect SUVR.

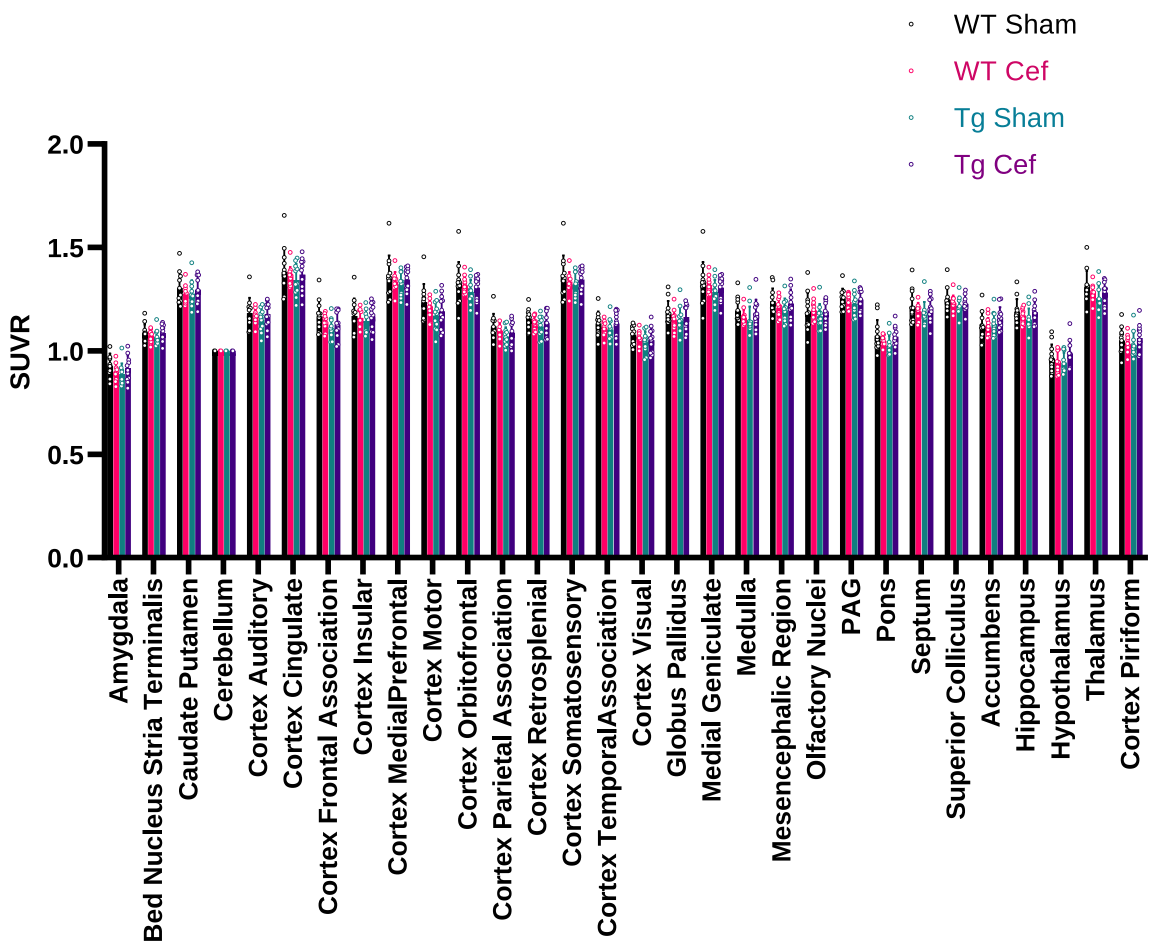

Figure S1: There were no ceftriaxone effects on FDG-PET in any VOI. Ceftriaxone did not affect SUVR in any brain region (3-way ANOVA, n = 12, $p > 0.05$).
